# Incidence trends and survival of metastatic prostate cancer with bone and visceral involvement: 2010-2019 surveillance, epidemiology, and end results

**DOI:** 10.3389/fonc.2023.1201753

**Published:** 2023-08-03

**Authors:** Gaohaer Kadeerhan, Bo Xue, Xiao-Lin Wu, Wei-Nan Chen, Dong-Wen Wang

**Affiliations:** ^1^ National Cancer Center/National Clinical Research Center for Cancer/Cancer Hospital & Shenzhen Hospital, Chinese Academy of Medical Sciences and Peking Union Medical College, Shenzhen, China; ^2^ Urology and Lithotripsy Center, Peking University People's Hospital, Beijing, China

**Keywords:** prostate cancer, bone metastasis, visceral metastasis, incidence, survival, SEER

## Abstract

**Background:**

The incidence of prostate cancer (PCa) has continued to increase since the US Preventive Services Task Force (USPSTF) recommendations against prostate-specific antigen (PSA)-based screening for all men in 2012, approximately half of additional diagnosed cases are advanced-stage, including regional PCa and metastatic PCa (mPCa). It is very important to investigate the shift in mPCa incidence and mPCa-related mortality risk, as the survival of mPCa remains poor.

**Objective:**

To investigate the incidence temporal trend of mPCa stratified by metastatic site, including bone and visceral metastatic involvement, and potential survival improvements.

**Materials:**

Based on the recently released Surveillance, Epidemiology, and End Results (SEER) data (2010-2019), the age-adjusted incidence rates of mPCa with bone and visceral involvement with annual percentage changes (APCs) were assessed by a joinpoint regression model in men aged 45 years and older by age and race groups, and potential recent improvements in overall survival (OS) and cancer-specific survival (CSS) were estimated by the Kaplan−Meier method and Cox regression model.

**Results:**

From 2010 to 2019, a total of 19081 (84.8%) and 3413 (15.2%) mPCa patients with bone and visceral involvement, respectively, were recorded in the SEER database. Considering all races and age groups, the incidence rate of mPCa with bone metastasis remained stable during 2017-2019 (APC, 0.9%; *p*=0.421) after increasing during 2010-2017 (APC, 5.8%; *p*<0.001). For visceral metastasis, the incidence rate increased by 12.3% (*p*<0.001) per year from 2010-2019. Non-Hispanic Black men have higher incidence rates than other populations, and the Non-Hispanic Black to Non-Hispanic White incidence rates ratios of mPCa declined with the greater increasing pace of incidence of Non-Hispanic White men. There was a slight improvement in both OS and CSS among men with bone and visceral metastasis involvement when comparing the 2013-2016 period to the pre-2013 period.

**Conclusion:**

Our findings show that the incidence of mPCa with bone and visceral involvement has increased in recent years and that there has been a potential improvement in survival. Future efforts are still needed to watch closely if the rising incidence trends continue.

## Introduction

The past three decades of prostate-specific antigen (PSA) screening have led to most prostate cancer (PCa) patients being discovered in the local or regional stages, and leading to a substantial reduction in PCa mortality (1). However, contradictory outcomes of two large trials that evaluated the impact of pupulation-based PSA screening (2, 3) and the considerable overdiagnosis and overtreatment resulted in recommendations against PSA screening for men over 75 years in 2008 and for all men in 2012 by the United States Preventive Services Task Force (USPSTF). With the decline in PSA screening, the PCa incidence has continued to rise by 3% per year from 2014-2019, with half of the additional diagnosed cases being advanced-stage, including regional-stage and metastatic PCa (mPCa) (4, 5). As the 5-year relative survival rate for mPCa is only 31%, it is very important to investigate the incidence pattern of mPCa and mPCa-related mortality risk.

Of note, mPCa patients present with different metastasis sites at diagnosis. Bone metastasis represents the most common location of mPCa, followed by more aggressive visceral involvement, including lung metastasis and liver metastasis (6, 7). Two prior studies based on population data demonstrated increased incidence rates of overall mPCa in recent years (4, 8). However, the incidence rates of specific metastatic sites of mPCa are unknown. Influenced by the pathobiology of the disease, the metastatic sites of mPCa have different prognostic impacts and also show differential responses to treatment (6, 9, 10). Although a small reduction in the risk of death was found in patients with newly diagnosed mPCa in recent years (11), given the change in screening policy and recent improvements in the treatment landscape of mPCa, the temporal trends of incidence and survival in mPCa patients with different metastasis sites still require population-based exploration.

Thus, in this study, we investigated the incidence rates and temporal trends of mPCa with bone and visceral involvement from 2010 to 2019 using the recently updated Surveillance Epidemiology and End Results (SEER) database. In addition, we assessed the overall survival (OS) and cancer-specific survival (CSS) of mPCa patients with bone and visceral involvement in recent years.

## 2 Materials and methods

### 2.1 Patient data sources

The mPCa cases diagnosed from 2010-2019 in the SEER database were selected (SEER 17, released April 2022, based on the November 2021 submission, accessed Nov 3, 2022). The inclusion criteria for patients were as follows: 1) PCa (ICD-O-3 code 61.9, histologic code 8140); 2) aged 45 years and older; 3) PCa was the only malignancy; and 4) referred to as distant mPCa or M1 stage according to the Combined Summary Stage or M1 AJCC stage, respectively.

Data were all publicly available and thus exempt from Institutional Review Board approval.

### 2.2 mPCa metastatic site definition and study variables

Combined Mets sites were noted as different metastasis sites in SEER, including bone, brain, liver, lung, distant lymph node and other sites. Cases were categorized into bone (with or without distant lymph node) or visceral (liver, lung and other sites with or without bone metastasis) metastasis involvement. The patients grouping information is presented in **Supplementary Table 1S**.

In addition, patient information, including age, race, diagnosis year, PSA, Gleason score, and treatment for surgery, radiotherapy, chemotherapy and systemic therapy, was included. Patient age at diagnosis was divided into two groups: 45-74 years and 75 years or older. Race was divided into three groups: non-Hispanic White, non-Hispanic Black, and other race.

### 2.3 Statistical analysis

The age-adjusted incidence rates of mPCa (standardized to the 2000 US population) were calculated using SEER*Stat software (version 8.4.0). The temporal trends in age-adjusted incidence rates of mPCa were analyzed using joinpoint regression analysis. The annual percentage changes (APCs) in incidence with 95% confidence intervals (CIs) were calculated by Joinpoint Regression Analysis software (version 4.9.1) (12). The model selects the best-fitting log-linear regression model to identify the time points when the APCs changed significantly. The tests of significance use a Monte Carlo permutation method. When the *p* value of the corresponding APC was less than 0.05, the trends were considered to be increasing or decreasing; otherwise, the trends were considered stable.

The non-Hispanic Black men or other races men to non-Hispanic White men differences in the mPCa incidence rates over time were investigated by computing incidence rate ratios (IRRs) and 95% CIs (13). For IRR variation over time, we also utilized the joinpoint regression method and visualized the results using the ggplot2 package.

We estimated the OS and CSS of mPCa patients with bone and visceral involvement. OS was defined as the time from mPCa diagnosis to all-caused death. CSS was defined as the time from mPCa diagnosis to prostate cancer-related death. Based on the recent updated SEER Follow-up Cut-off Date (December 31, 2019), patients diagnosed from 2010 to 2016 with a follow-up time of 3 years or more were included. Kaplan−Meier curves and a log-rank test were used to analyse the OS and CSS among age and race groups, and Cox proportional hazards regressions were used to test the survival improvements between 2013-2016 and 2010-2012 by R software (version 4.0.2). All statistical tests were two-sided, and *p* < 0.05 was considered statistically significant.

## 3 Results

### 3.1 Study cases

From 2010 to 2019, a total of 22494 patients with distant mPCa were diagnosed in men aged 45 years and older in the SEER database, of whom 19081 (84.8%) had bone involvement and 3413 (15.2%) had visceral involvement. The age, race, and other characteristic distributions of mPCa patients are presented in **Supplementary Table 2S**. Overall, both bone and visceral involvement mPCa patients had the same age distribution: 63.2% of patients were 45-74 years, and 36.8% of patients were ≥75 years. In patients with bone metastasis, 12017 (63.0%) were non-Hispanic White, 3178 (16.7%) were non-Hispanic Black, and 3886 (20.3%) were other races. In patients with visceral metastasis, 1955 (57.3%) were non-Hispanic White, 702 (20.6%) were non-Hispanic Black, and 756 (22.1%) were other races.

### 3.2 Overall incidence rates and temporal trends of mPCa

**Figure 1:**
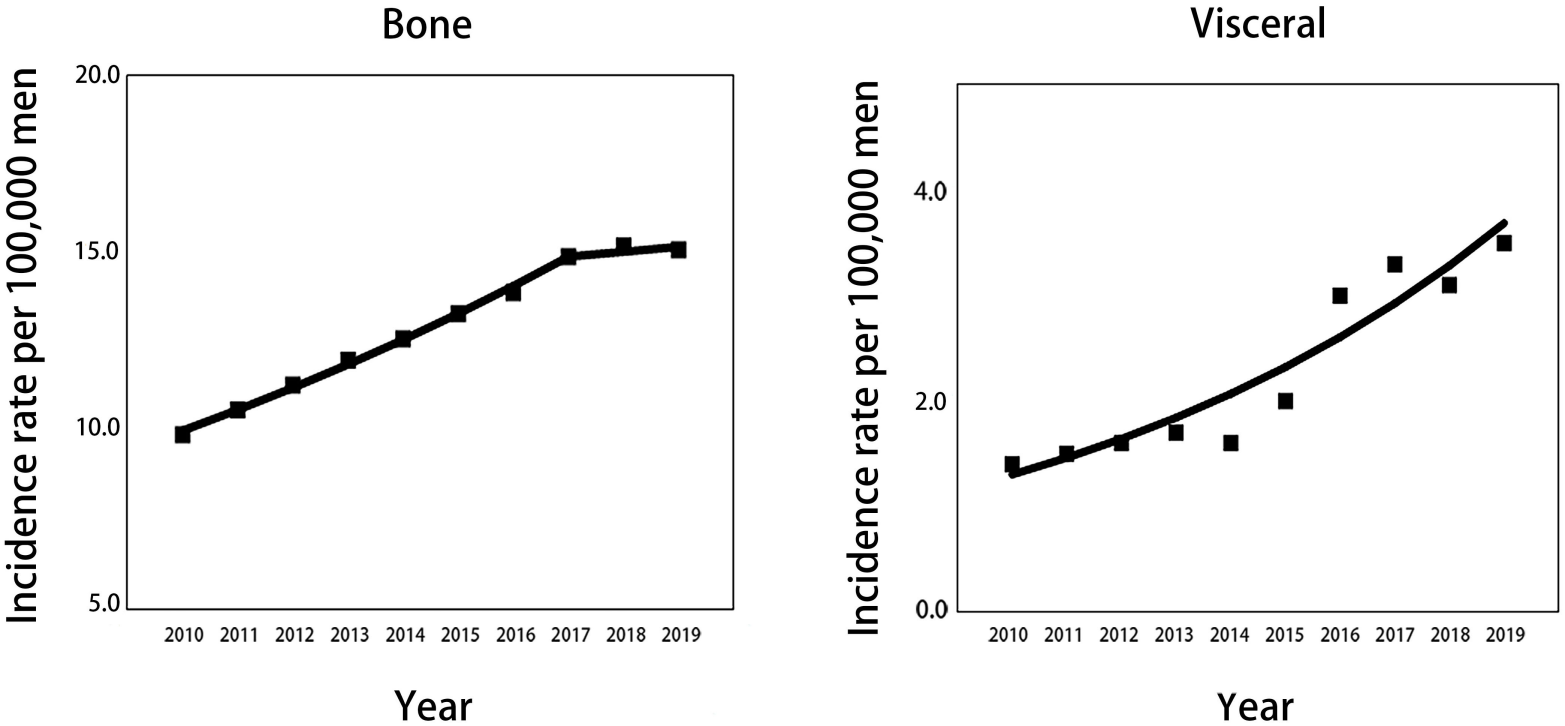
Trends in annual age-standardized incidence rates of mPCa with bone and visceral metastasis involvement for all patients aged 45 years and older. Solid lines represent joinpoint modelled rates, and block symbols represent observed rates. mPCa, metastatic prostate cancer.

Considering all races aged 45 and older, the incidence rate per 100000 men of mPCa with bone metastasis increased significantly from 9.9 (95% CI: 9.3-10.4) in 2010 to 14.9 (95% CI: 14.3-15.6) in 2017 and then remained stable to 15.1 (95% CI: 14.5-15.7) in 2019. For visceral metastasis, incidence increased throughout the study period, from 1.4 (95% CI: 1.2-1.7) in 2010 to 3.5 (95% CI: 3.2-3.8) in 2019 (**Table 1**; **Figure 1**). According to joinpoint regression analysis (**Table 2**), incidence rates for bone metastasis significantly increased by 5.8% (95% CI: 5.3%-6.3%, *p*<0.001) per year from 2010 to 2017 and then stabilized from 2017 to 2019 (*p*=0.421). Incidence rates for visceral metastasis continued to rise significantly by 12.3% (95% CI: 8.3%-16.3%, *p*<0.001) per year during 2010-2019.

**Table 1:** Age-standardized incidence rates of mPCa with bone and visceral metastasis involvement in the SEER database 2010-2019^a^.

Year	Bone, n=19081		Visceral, n=3413	
	No. of patients	Incidence (95% CI)	No. of patients	Incidence (95% CI)
**2010**	1269	9.9 (9.3-10.4)	186	1.4 (1.2-1.7)
**2011**	1413	10.6 (10.0-11.2)	189	1.5 (1.2-1.7)
**2012**	1538	11.3 (10.7-11.9)	222	1.6 (1.4-1.9)
**2013**	1679	12.0 (11.4-12.6)	239	1.7 (1.4-1.9)
**2014**	1831	12.6 (12.0-13.2)	238	1.6 (1.4-1.8)
**2015**	1969	13.3 (12.7-14.0)	285	2.0 (1.7-2.2)
**2016**	2116	13.9 (13.3-14.6)	462	3.0 (2.8-3.3)
**2017**	2339	14.9 (14.3-15.6)	508	3.3 (3.0-3.6)
**2018**	2440	15.2 (14.6-15.8)	506	3.1 (2.9-3.4)
**2019**	2487	15.1 (14.5-15.7)	578	3.5 (3.2-3.8)

a Rates are per 100 000 and age-adjusted to the 2000 US Standard Population standard.

CI, confidence interval; mPCa, metastatic prostate cancer.

**Table 2:** Trends in annual age-standardized mPCa incidence rates by metastasis involvement sites, race, and age in the SEER database, 2010-2019.

	Age	Trends 2010-2019		
		Segment years ^a^	APC (95%CI)	*p*
All races				
Bone	45-74	2010-2019	4.3 (3.5-5.2)	< 0.001
	≥75	2010-2019	5.6 (4.2-7.0)	< 0.001
	all	2010-2017	5.8 (5.3-6.3)	< 0.001
		2017-2019	0.9 (-1.8-3.7)	0.421
Visceral	45-74	2010-2019	11.8 (8.8-14.9)	< 0.001
	≥75	2010-2019	13.4 (7.4-19.7)	< 0.001
	all	2010-2019	12.3 (8.3-16.3)	<0.001
Non-Hispanic White				
Bone	45-74	2010-2019	5.0 (3.7-6.3)	<0.001
	≥75	2010-2015	9.6 (5.8-13.5)	0.001
		2015-2019	3.0 (-1.1-1.9)	0.116
	all	2010-2017	6.9 (5.9-7.9)	<0.001
		2017-2019	0.7 (-5.3-7.1)	0.782
Visceral	45-74	2010-2019	12.1 (8.6-15.6)	<0.001
	≥75	2010-2019	13.4 (7.1-20.1)	0.001
	all	2010-2019	13.5 (9.1-17.9)	<0.001
Non-Hispanic Black				
Bone	45-74	2010-2019	2.7 (1.2-4.1)	0.002
	≥75	2010-2019	2.8 (1.0-3.6)	0.007
	all	2010-2019	2.7 (1.5-4.0)	0.001
Visceral	45-74	2010-2019	8.7 (4.8-12.9)	0.001
	≥75	2010-2019	10.7 (2.0-20.1)	0.021
	all	2010-2019	9.5 (4.9-14.3)	0.001
Other				
Bone	45-74	2010-2019	3.5 (2.2-4.8)	<0.001
	≥75	2010-2019	4.2 (2.5-5.9)	<0.001
	all	2010-2019	3.9 (3.0-4.8)	<0.001
Visceral	45-74	2010-2019	10.3 (4.3-16.6)	0.004
	≥75	2010-2019	14.0 (7.4-21.0)	0.001
	all	2010-2019	13.4 (8.6-18.3)	<0.001

a Segments determined by the joinpoint model.

APC, annual percentage change; CI, confidence interval; mPCa, metastatic prostate cancer.

### 3.3 Age- and race-specific incidence rates and temporal trends of mPCa

**Figure 2:**
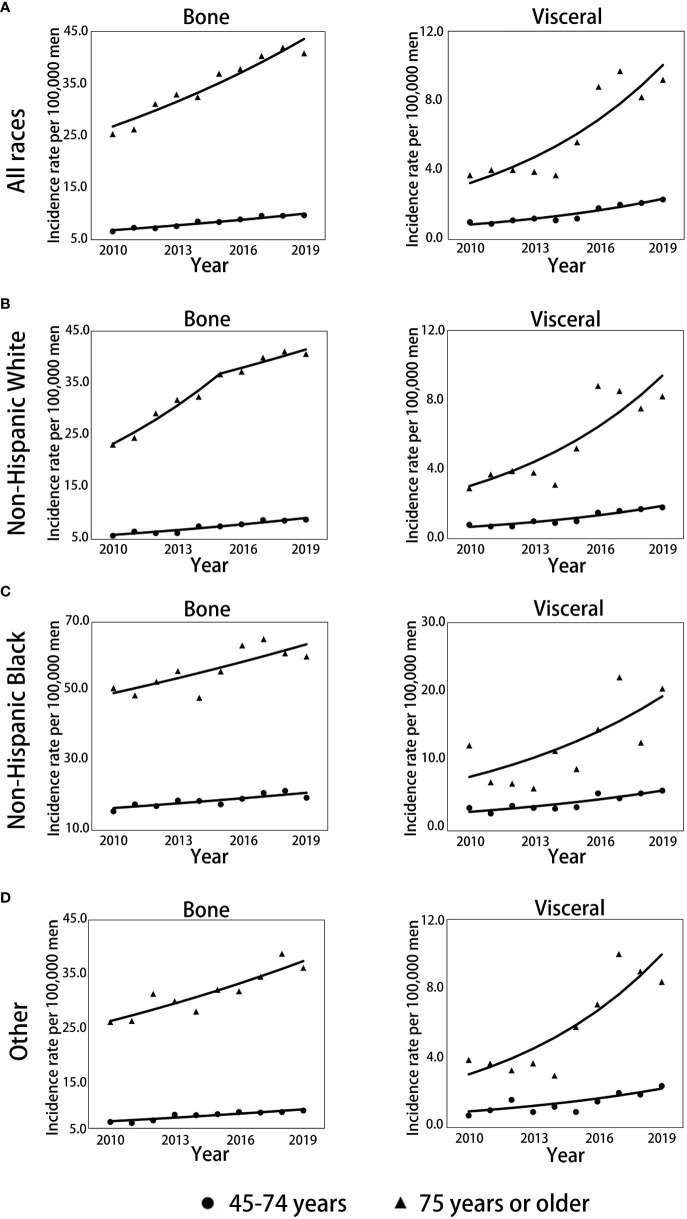
Trends in annual age-standardized incidence rates of mPC with bone and visceral metastasis involvement stratified by race and age. **(A)** Trends for all races combined for men aged 45-74 years and 75 years or older. **(B)** Trends for non-Hispanic White men aged 45-74 and 75 years or older. **(C)** Trends for non-Hispanic Black men aged 45-74 and 75 years or older. **(D)** Trends for men of other races aged 45-74 and 75 years or older. Solid lines represent joinpoint modelled rates, and symbols represent observed rates. mPCa, metastatic prostate cancer.

The trends over time for the incidence rates per 100000 men for mPCa cases with bone or visceral metastasis by age and race groups are depicted in **Figure 2**; **Table 2** and **Supplementary Table 3S.** For all races in mPCa cases, the incidence of bone metastasis and visceral metastasis for patients aged 45-74 years and ≥75 years both significantly increased from 2010 to 2019 (**Figure 2A**). In men aged 45-74 years (**Table 2**; **Supplementary Table 3S**), the mPCa with bone involvement incidence rate increased from 6.6 (95% CI: 6.2-7.1) in 2010 to 9.7 (95% CI: 9.2-10.2) in 2019 (APC, 4.3%; *p*<0.001), and the mPCa with visceral involvement incidence rate increased from 1.0 (95% CI: 0.8-1.2) in 2010 to 2.3 (95% CI: 2.1-2.6) in 2019 (APC, 11.8%; *p*<0.001). In men aged 75 years or older (**Table 2**; **Supplementary Table 3S**), the incidence rate for mPCa with bone involvement increased from 25.3 (95% CI: 23.0-27.7) in 2010 to 40.8 (95% CI: 38.2-43.5) in 2019 (APC, 5.6%; *p*<0.001), and the incidence rate for mPCa with visceral involvement increased from 3.7 (95% CI: 2.9-4.7) in 2010 to 9.2 (95% CI: 8.0-10.5) in 2019 (APC, 13.4%; *p*<0.001).

The incidence rates of bone or visceral metastasis involvement in non-Hispanic Black men were generally higher than those in non-Hispanic White and other races men during 2010-2019 (**Figures 2B–D**; **Supplementary Table 3S**). Although the incidence temporal trends presented continued increases for bone or visceral metastasis involvement in non-Hispanic White, non-Hispanic Black, and other race men stratified by age groups throughout the study period, the incidence patterns of bone metastasis in non-Hispanic White men aged 75 years or older remained stable from 2015 to 2019 (APC: 3.0%, *p*=0.116, **Figure 2B**; **Table 2**) after significantly increasing pre-2015 (APC: 9.6%, *p*=0.001, **Figure 2B**; **Table 2**), and the pace of the increase in bone and visceral metastasis was slower in non-Hispanic Black men than in non-Hispanic White and other race men (**Table 2**).

### 3.4 IRRs of non-Hispanic Black to non-Hispanic White and other races to non-Hispanic White

**Figure 3:**
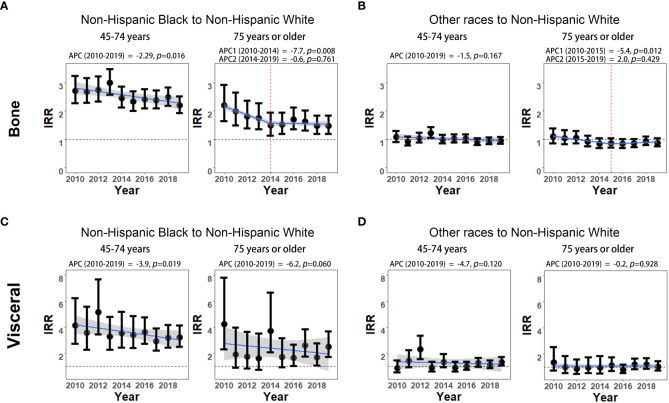
Non-Hispanic Black to non-Hispanic White and other races to non-Hispanic White incidence rate ratios (IRRs) of mPCa with bone and visceral metastasis involvement. **(A)** Non-Hispanic Black to non-Hispanic White IRRs of mPCa with bone metastasis involvement. **(B)** Other races to non-Hispanic White IRRs of mPCa with bone metastasis involvement. **(C)** Non-Hispanic Black to non-Hispanic White IRRs of mPCa with visceral metastasis involvement. **(D)** Other races to non-Hispanic White IRRs of mPCa with visceral metastasis involvement. IRR, incidence rate ratio; mPCa, metastatic prostate cancer.

The non-Hispanic Black to non-Hispanic White IRRs of bone metastasis significantly declined from 2.72 (95% CI: 2.28-3.24) to 2.20 (95% CI: 1.93-2.51) among men aged 45-74 years (APC, -2.29%; *p*=0.016). The IRR disparities between non-Hispanic Black and non-Hispanic White also decreased from 2.20 (95% CI: 1.65-2.93) to 1.49 (95% CI: 1.14-1.94) during 2010-2014 (APC, -7.7%; *p*=0.008) and then remained stable during 2014-2019 (APC, -0.6%; *p*=0.761) among men aged 75 years or older (**Figure 3A**; **Supplementary Table 4S**). Although the IRRs of visceral metastasis are generally higher than that of bone metastasis between non-Hispanic Black and non-Hispanic White, the IRR disparities of visceral metastasis between these two groups showed a significant decline from 4.13 (95% CI: 2.73-6.24) to 3.22 (95% CI: 2.50-4.15) among men aged 45-74 years during 2010 to 2019 (APC, -3.9%; *p*=0.019). However, in the age group of 75 years or older, the declining trends in IRRs of visceral metastasis did not reach statistical significance (APC, -6.2%; *p*=0.060, **Figure 3C**; **Supplementary Table 4S**). No significant changes in the IRRs of bone and visceral involvement were observed between other races and non-Hispanic White mPCa men in either age group, except for a significant decline in bone metastasis rates among the 75 years or older age group during 2010-2015 (**Figures 3B, D**; **Supplementary Table 5S**).

### 3.5 OS and CSS of mPCa with bone and visceral metastasis involvement

A total of 13636 and 11629 mPCa patients met the OS and CSS analyses, respectively. The median OS and CSS were 31 months (95% CI: 31-32) and 34 months (95% CI: 33-35) for men with bone metastases and 20 months (95% CI: 19-22) and 22 months (95% CI: 20-24) for men with visceral metastases (all *p* values <0.001, **Supplementary Figure 1S**).

To examine the mPCa survival improvement, OS and CSS across time periods (2013-2016 v.s. 2010-2012) were compared (**Supplementary Table 6S**). In terms of OS, comparing 2013-2016 with 2010-2012, a 3% and a 4% decreased risk of death were found in non-Hispanic White (HR: 0.97, 95% CI: 0.95-0.99, *p*<0.001) and non-Hispanic Black patients (HR: 0.96, 95% CI: 0.93-0.99, *p=*0.009) aged 45-74 years with bone metastasis involvement, whereas no statistically significant differences in OS were found between the two time periods among the race subgroups aged 75 years or older with bone metastasis involvement. For visceral metastasis involvement, both a 7% decreased risk of death was presented in non-Hispanic Black patients aged 45-74 years (HR: 0.93, 95% CI: 0.87-0.99, *p=*0.032) and in non-Hispanic White patients aged 75 years or older (HR: 0.93, 95% CI: 0.88-0.97, *p=*0.003) during 2013-2016 compared with 2010-2012 for OS.

In terms of CSS (**Supplementary Table 6S**), comparing 2013-2016 with 2010-2012, significant decreasing trends were displayed in all races subgroups aged 45-74 years (all *p* values<0.05) and in non-Hispanic White patients aged 75 years or older with bone metastasis involvement (*p*<0.001). When considering patients with visceral metastasis involvement, comparing 2013-2016 with 2010-2012, a significantly decreased risk of death was shown for CSS in non-Hispanic White and non-Hispanic Black patients aged 45-74 years and 75 years or older (all *p* values<0.05).

## 4 Discussion

In this population-based study of SEER data, we noted an increase in the incidence rates of mPCa with bone and visceral metastasis involvement during 2010-2019, and more pronounced OS and CSS improvements were shown in 2013-2016 than in 2010-2012 for mPCa with bone and visceral metastasis involvement.

The rising incidence of distant stage has recently been increasingly reported in PCa (4, 8, 12, 13). Although multiple factors, including family history, environmental exposures, and excess body weight, are associated with fatal PCa risk (14–16), it is unlikely to lead to alterations in epidemiological patterns of cancers in a short time. The major policy shift proposed by the USPSTF recommendation is to against routine population-based PSA testing since 2012 (17), which may help to explain such changes in mPCa incidence trends. Prior studies have reported that PSA testing rates have fallen, followed by USPSTF recommendation (18). Consequently, more than doubled distant-stage PCa has been diagnosed (19). Among them, patients with bone and visceral involvement are not negligible, and their incidence rates have not been fully investigated. In our study, we utilized joinpoint regression analysis to examine the temporal trends in age-adjusted incidence rates of mPCa with bone and visceral metastasis. Our findings indicated an increase in the incidence rates of mPCa with bone and visceral metastasis over the study period. However, it is important to note that the incidence of bone metastasis remained stable after 2017. These results provide valuable insights into the specific years when significant changes occurred in mPCa incidence rates. However, it is essential to recognize that our analysis represents a simplified account of the observed temporal trends. Furthermore, since the USPSTF only recently updated its recommendation in 2018 to advocate informed decision making for men aged 55-69 years (20), further long-term population-based studies are necessary to elucidate whether the observed increased incidence patterns of mPCa with bone and visceral metastasis will persist in the future.

Our study reported a significantly increased incidence of mPCa with bone metastasis and visceral metastasis in both age groups (≥75 years vs 45-74 years). Of note, the incidence rates in men aged 75 years or older were higher, and the increased pace was steeper than that in men aged 45-74 years (APC of bone metastasis: 5.6% vs 4.3%, APC of visceral metastasis: 13.4% vs 11.8%). This is in line with the PCa incidence increasing with age (14). On the other hand, the reason for this disparity is thought to reflect that there was an earlier and greater reduction in PSA screening in men aged 75 years or older (21). A previous study looking at overall mPCa in SEER data up to 2018 presented the rise in incidence of mPCa in younger aged men predominantly observed in the latter years (2015-2018) (4). This age disparity in the mPCa incidence pattern may bring challenges to clinical treatments in the real world, so future studies with stringent surveillance are still needed.

Racial differences were found in bone and visceral metastasis incidence trends in our analysis. In non-Hispanic Black men, the bone and visceral metastasis incidences were higher than those in other populations. Reasons for these disparities are not entirely clear; however, racial disparities in PSA testing prevalence, lifestyle factors, biological susceptibilities, and healthcare access may partially explain these patterns (14, 22–25). Non-Hispanic Black men exhibit lower PSA testing rates and tend to have more prevalent obesity and cigarette smoking. Furthermore, evidence has shown that non-Hispanic Black men harbor multiple susceptibility variants of PCa. Nevertheless, in line with previous reports (8), a substantial decline in the racial disparity between non-Hispanic Black and non-Hispanic White men in both bone and visceral metastasis involvement have also been noted in our study. This decline in racial disparity largely confined to men aged 45-74, which aligns with the observed increase in mPCa incidence among non-Hispanic White men in our study. Identification of the precise factors underlying these patterns requires further investigation.

The increasing incidence of mPCa in recent years has received public health attention given the premature mortality associated with it. In the present study, we analyzed survival in men diagnosed with mPCa with bone and visceral involvement in the last decade. Similar to the previous SEER-based survival analysis (6, 26), visceral involvement conferred worse survival than bone involvement. Survival rate differences between non-Hispanic Black men and non-Hispanic White men were found only in bone metastasis involvement in men aged 45-74 years for OS and in men aged 75 years or older for CSS, although non-Hispanic Black men showed generally lower median survival times. These observations are associated with different rates of treatment among racial patients and imply that treating mPCa is still full of difficulties and challenges (27).

We did find significant differences in OS and CSS between 2010-2012 and 2013-2016. Although the study variables may not fully capture all relevant clinical factors that could impact outcomes, they should be sufficient to preliminary confirm the potential slight improvements in OS and CSS in recent years. This is supported by the similarity of our results to those of two recently published articles (27, 28). The numerous advances in mPCa treatment have led to multiple new drug approvals in recent years, which might in part explain the survival improvements in our results (11, 28). However, not all patients can receive rational chemotherapy in real-world clinical practice. In addition, optimal sequencing and combination of the various agents in real therapies are currently unknown. Collectively, these results provide possible explanations for the small improvement in survival outcomes.

Regardless of the underlying cause, an increase in the incidence of mPCa that observed in our study does not indicate a need to change screening practices. The decision to implement population- based PSA screening involves a complex evaluation of the overall risk versus benefit, and taking into consideration various factors that affect the overall health of the population. However, based on SEER data available to us, it is noteworthy that we have observed a sustained rise in the incidence rate of mPCa across in different age and race groups. This finding is novel and significant, warranting further evaluation and investigation. As the metastatic sites of mPCa have different prognostic impacts, a comprehensive understanding of the epidemiological patterns of mPCa, combined with in-depth exploration of the biological mechanisms associated with mPCa patients outcomes, is essential for the development of effective targeted treatment strategies in future too, though there have been several studies reporting on the potential theragnostic window in high-risk prostate cancer (29).

Our study has some limitations. First, the data in our study are not delay adjusted, which may have underestimated the increasing trends for our results. Second, SEER data lack full information on diagnostic techniques, number of metastasis sites, body mass index, and lines of treatment, so we cannot make causal inferences. Third, in 2018, the USPSTF recommendation was modified as informed decision-making in men aged 55-69 years, and the incidence pattern linkage to this screening change needs further observation. Fourth, the follow-up duration in our comparison groups, particularly for recent cases, remains relatively short, therefore, the survival pattern needs further longer-term estimation. Finally, we included cases that involve multiple metastatic sites for the full extent of disease burden. However, due to the complexities of managing these cases in the real world and the challenges associated with prognosis assessment, further in-depth research will be needed in the future to analyze the incidence and survival of these mPCa cases with multiple metastatic sites. Collectively, the more studies beyond the SEER data and alternative methods may necessary to support and confirm our results in future.

## 5 Conclusions

Our study suggests increasing incidence rates of mPCa with bone and visceral metastasis involvement in recent years after 2012 USPTF recommendations against routine PSA testing for all men. The rising trends were seen in all age groups and in all race groups. Although non-Hispanic Black men harbor higher incidence rates than other populations, the non-Hispanic Black to non-Hispanic White IRRs declined with the greater increasing pace of incidence of non-Hispanic White men. There has been a potential improvement in the survival of mPCa patients with bone and visceral involvement in recent years. Further studies are needed to clarify whether this increasing pattern continues.

## Data availability statement

The original contributions presented in the study are included in the article/**Supplementary Material.** Further inquiries can be directed to the corresponding author.

## Ethics statement

Ethical review and approval were not required for the study on human participants in accordance with the local legislation and institutional requirements. Written informed consent for participation was not required for this study in accordance with the national legislation and the institutional requirements.

## Author contributions

D-WW had full access to all the data in the study and takes responsibility for the integrity of the data and the accuracy of the data analysis. Study concept and design: D-WW and KG. Acquisition of data: KG. Analysis and interpretation of data: GK, BX, X-LW, and W-NC. Drafting of the manuscript: GK Critical revision of the manuscript for important intellectual content: BX, X-LW, W-NC, and D-WW. Statistical analysis: GK and BX. Supervision: D-WW. All authors contributed to the article and approved the submitted version.
