# Supplementary material for: Incidence trends and survival of metastatic prostate cancer with bone and visceral involvement: 2010-2019 surveillance, epidemiology, and end results
Source: Front Oncol. 2023 Aug 3;13:1201753. doi: 10.3389/fonc.2023.1201753 (PMC10435983; doi:10.3389/fonc.2023.1201753)
Supplement: Supplementary file 1 [file DataSheet_1.docx]

# Supplementary Tables

# **S1** Table. mPCa metastatic site definition and grouping information

|  | **Number** | **Combined Summary Stage** | **Derived SEER Combined M** | **SEER Combined Mets at DX-bone** | **SEER Combined Mets at DX-liver** | **SEER Combined Mets at DX-lung** | **SEER Combined Mets at DX-brain** | **Mets at DX-Distant LN (2016+)** | **Mets at DX-Other**  **(2016+)** |
| --- | --- | --- | --- | --- | --- | --- | --- | --- | --- |
| **Presense of Bone Metastases** | **19081** | Distant | M1a/M1b | Yes | No | No | No | Yes/No/  Unknown | No |
|  |  |  |  |  |  |  |  |  |  |
| Presense of Visceral Metastases | **3413** | Distant | M1a/M1b/M1c | Yes/No | Yes | Yes/No  /Unknown | Yes/No/  Unknown | Yes/No/  Unknown | Yes/No  /Unknown |
|  |  |  |  |  | Yes/No/Unknown | Yes | Yes/No/  Unknown |  |  |

# **S2** Table. General characteristics distribution of mPCa patients with bone or visceral metastasis involvement

|  | **Total,**  **n=22494** | **Bone,**  **n=19081** | **Visceral,**  **n=3413** |
| --- | --- | --- | --- |
| **Age group** |  |  |  |
| 45-74 years | 14210 (63.2) | 12052 (63.2) | 2158 (63.2) |
| ≥75 years | 8284 (36.8) | 7029 (36.8) | 1255 (36.8) |
| **Race and ethnicity** |  |  |  |
| Non-Hispanic White | 13972 (62.1) | 12017 (63.0) | 1955 (57.3) |
| Non-Hispanic Black | 3880 (17.2) | 3178 (16.7) | 702 (20.6) |
| Other | 4642 (20.7) | 3886 (20.3) | 756 (22.1) |
| **PSA (ng/ml)** | 98.0 (30.9-98.0) | 98.0 (30.0-98.0) | 98.0 (37.3-98.0) |
| **Gleason score** |  |  |  |
| ≤7 | 2333 (10.4) | 2095 (11.0) | 238 (7.0) |
| >7 | 12797 (56.9) | 11201 (58.7) | 1596 (46.8) |
| Not reported | 7364 (32.7) | 5785 (30.3) | 1579 (46.3) |
| **Surgery** |  |  |  |
| Yes | 2410 (10.7) | 2017 (10.6) | 393 (11.5) |
| No/Unknown | 20084 (89.3) | 17064 (89.4) | 3020 (88.5) |
| **Radiation therapy** |  |  |  |
| Yes | 5461 (24.3) | 4670 (24.5) | 791 (23.2) |
| No/Unknown | 17033 (75.7) | 14411 (75.5) | 2622 (76.8) |
| **Chemotherapy** |  |  |  |
| Yes | 3567 (15.9) | 2830 (14.8) | 737 (21.6) |
| No/Unknown | 18927 (84.1) | 16251 (85.2) | 2676 (78.4) |
| **Systemic therapy** |  |  |  |
| Yes | 2926 (13.0) | 2377 (12.5) | 549 (16.1) |
| No/Unknown | 19568 (87.0) | 16704 (87.5) | 2864 (83.9) |

PSA, prostate-specific antigen

**S3 Table. Yearly variations in mPCa age-standardized incidence rates stratified by metastasis involvement sites, race, and age** **in SEER database 2010-2019 ^a^**

|  | **Age** | **Year** | | | | | | | | | |
| --- | --- | --- | --- | --- | --- | --- | --- | --- | --- | --- | --- |
|  |  | **2010** | **2011** | **2012** | **2013** | **2014** | **2015** | **2016** | **2017** | **2018** | **2019** |
| **All races** |  |  |  |  |  |  |  |  |  |  |  |
| Bone | 45-74 | 6.6 (6.2-7.1) | 7.3 (6.9-7.8) | 7.2 (6.7-7.7) | 7.6 (7.2-8.1) | 8.5 (8.0-9.0) | 8.4 (7.9-8.9) | 8.9 (8.5-9.4) | 9.6 (9.1-10.1) | 9.6 (9.1-10.1) | 9.7 (9.2-10.2) |
|  | ≥75 | 25.3 (23.0-27.7) | 26.2 (23.9-28.6) | 31.1 (28.6-33.7) | 32.9 (30.4-35.6) | 32.4 (30.0-35.1) | 36.9 (34.3-39.6) | 37.8 (35.2-40.5) | 40.3 (37.7-43.1) | 41.9 (39.2-44.7) | 40.8 (38.2-43.5) |
|  | all | 9.9 (9.3-10.4) | 10.6 (10.0-11.2) | 11.3 (10.7-11.9) | 12.0 (11.4-12.6) | 12.6 (12.0-13.2) | 13.3 (12.7-14.0) | 13.9 (13.3-14.6) | 14.9 (14.3-15.6) | 15.2 (14.6-15.8) | 15.1 (14.5-15.7) |
| Visceral | 45-74 | 1.0 (0.8-1.2) | 0.9 (0.8-1.1) | 1.1 (1.0-1.3) | 1.2 (1.0-1.4) | 1.1 (1.0-1.3) | 1.2 (1.0-1.4) | 1.8 (1.6-2.1) | 2.0 (1.7-2.2) | 2.1 (1.9-2.3) | 2.3 (2.1-2.6) |
|  | ≥75 | 3.7 (2.9-4.7) | 4.0 (3.1-5.0) | 4.0 (3.1-5.0) | 3.9 (3.1-4.9) | 3.7 (2.9-4.6) | 5.6 (4.6-6.8) | 8.8 (7.6-10.2) | 9.7 (8.4-11.1) | 8.2 (7.1-9.5) | 9.2 (8.0-10.5) |
|  | all | 1.4 (1.2-1.7) | 1.5 (1.2-1.7) | 1.6 (1.4-1.9) | 1.7 (1.4-1.9) | 1.6 (1.4-1.8) | 2.0 (1.7-2.2) | 3.0 (2.8-3.3) | 3.3 (3.0-3.6) | 3.1 (2.9-3.4) | 3.5 (3.2-3.8) |
| **Non-Hispanic White** |  |  |  |  |  |  |  |  |  |  |  |
| Bone | 45-74 | 5.7 (5.2-6.2) | 6.5 (6.0-7.1) | 6.2 (5.7-6.7) | 6.2 (5.7-6.7) | 7.5 (6.9-8.0) | 7.5 (6.9-8.0) | 7.9 (7.4-8.5) | 8.7 (8.1-9.3) | 8.6 (8.0-9.2) | 8.8 (8.2-9.4) |
|  | ≥75 | 23.2 (20.7-25.9) | 24.5 (21.9-27.2) | 29.2 (26.5-32.2) | 31.8 (29.0-34.9) | 32.4 (29.5-35.5) | 36.7 (33.7-40.0) | 37.2 (34.2-40.5) | 39.9 (36.8-43.2) | 41.1 (38.0-44.5) | 40.6 (37.6-43.9) |
|  | all | 8.7 (8.1-9.3) | 9.6 (9.0-10.3) | 10.2 (9.5-10.9) | 10.6 (10.0-11.3) | 11.8 (11.1-12.5) | 12.5 (11.8-13.3) | 13.0 (12.3-13.7) | 14.1 (13.4-14.8) | 14.2 (13.5-15.0) | 14.3 (13.6-15.0) |
| Visceral | 45-74 | 0.8 (0.6-1.0) | 0.7 (0.5-0.9) | 0.7 (0.5-0.9) | 1.0 (0.8-1.2) | 0.9 (0.7-1.1) | 1.0 (0.8-1.2) | 1.5 (1.2-1.7) | 1.6 (1.3-1.8) | 1.7 (1.5-2.0) | 1.8 (1.5-2.0) |
|  | ≥75 | 2.9 (2.0-3.9) | 3.7 (2.8-4.9) | 3.9 (3.0-5.1) | 3.8 (2.9-5.0) | 3.1 (2.3-4.2) | 5.2 (4.1-6.6) | 8.8 (7.4-10.5) | 8.5 (7.1-10.0) | 7.5 (6.2-9.0) | 8.2 (6.9-9.7) |
|  | all | 1.1 (0.9-1.4) | 1.2 (1.0-1.5) | 1.2 (1.0-1.5) | 1.5 (1.2-1.7) | 1.3 (1.1-1.5) | 1.7 (1.5-2.0) | 2.7 (2.4-3.1) | 2.8 (2.4-3.1) | 2.7 (2.4-3.1) | 2.9 (2.6-3.2) |
| **Non-Hispanic Black** |  |  |  |  |  |  |  |  |  |  |  |
| Bone | 45-74 | 15.5 (13.1-18.1) | 17.5 (15.1-20.2) | 17.0 (14.7-19.7) | 18.6 (16.3-21.2) | 18.5 (16.2-21.1) | 17.5 (15.2-19.9) | 19.1 (16.8-21.7) | 20.8 (18.5-23.4) | 21.4 (19.0-24.0) | 19.4 (17.2-21.8) |
|  | ≥75 | 51.0 (38.4-66.5) | 48.9 (36.8-63.8) | 52.8 (40.4-67.8) | 55.9 (43.3-71.1) | 48.2 (36.8-62.0) | 55.8 (43.6-70.2) | 63.3 (50.8-78.1) | 65.1 (52.5-79.9) | 61.0 (49.1-74.8) | 60.1 (48.4-73.7) |
|  | all | 21.6 (18.6-24.9) | 22.9 (20.0-26.2) | 23.2 (20.3-26.5) | 25.1 (22.1-28.4) | 23.7 (20.8-26.8) | 24.1 (21.3-27.2) | 26.8 (23.9-30.0) | 28.5 (25.5-31.7) | 28.2 (25.4-31.4) | 26.5 (23.7-29.4) |
| Visceral | 45-74 | 3.3 (2.2-4.6) | 2.5 (1.6-3.6) | 3.6 (2.5-4.9) | 3.3 (2.3-4.6) | 3.2 (2.3-4.5) | 3.4 (2.5-4.6) | 5.4 (4.2-6.8) | 4.7 (3.7-6.0) | 5.4 (4.2-6.8) | 5.8 (4.6-7.1) |
|  | ≥75 | 12.3 (6.7-20.8) | 7.0 (3.0-14.0) | 6.8 (2.9-13.5) | 6.1 (2.5-12.6) | 11.5 (6.4-19.1) | 8.9 (4.6-15.6) | 14.6 (8.9-22.6) | 22.1 (15.0-31.4) | 12.7 (7.6-19.9) | 20.5 (14.1-28.9) |
|  | all | 4.8 (3.5-6.5) | 3.3 (2.2-4.6) | 4.1 (3.0-5.6) | 3.8 (2.7-5.2) | 4.7 (3.4-6.2) | 4.4 (3.2-5.8) | 7.0 (5.6-8.7) | 7.7 (6.2-9.6) | 6.7 (5.3-8.3) | 8.3 (6.8-10.1) |
| **Other** |  |  |  |  |  |  |  |  |  |  |  |
| Bone | 45-74 | 6.2 (5.3-7.3) | 6.0 (5.1-7.0) | 6.5 (5.6-7.5) | 7.6 (6.7-8.7) | 7.5 (6.6-8.5) | 7.7 (6.8-8.7) | 8.1 (7.2-9.1) | 8.0 (7.1-9.0) | 8.1 (7.2-9.1) | 8.4 (7.6-9.4) |
|  | ≥75 | 25.4 (20.2-31.5) | 25.6 (20.6-31.6) | 30.8 (25.3-37.0) | 29.4 (24.3-35.4) | 27.4 (22.5-33.0) | 31.6 (26.5-37.4) | 31.3 (26.3-36.9) | 34.1 (29.1-39.8) | 38.5 (33.3-44.4) | 35.8 (30.8-41.3) |
|  | all | 9.5 (8.3-10.9) | 9.4 (8.3-10.7) | 10.7 (9.5-12.0) | 11.4 (10.2-12.7) | 11.0 (9.8-12.2) | 11.9 (10.7-13.1) | 12.1 (10.9-13.3) | 12.5 (11.4-13.8) | 13.4 (12.2-14.6) | 13.2 (12.0-14.4) |
| Visceral | 45-74 | 0.7 (0.4-1.1) | 1.0 (0.6-1.4) | 1.6 (1.2-2.2) | 0.9 (0.6-1.4) | 1.2 (0.8-1.6) | 0.9 (0.6-1.2) | 1.5 (1.1-1.9) | 2.0 (1.5-2.5) | 1.9 (1.5-2.4) | 2.4 (2.0-3.0) |
|  | ≥75 | 3.9 (2.1-6.7) | 3.7 (2.0-6.4) | 3.3 (1.7-5.8) | 3.7 (2.0-6.1) | 3.0 (1.5-5.2) | 5.8 (3.8-8.6) | 7.1 (4.9-10.1) | 10.0 (7.3-13.2) | 9.0 (6.6-12.1) | 8.4 (6.1-11.3) |
|  | all | 1.3 (0.9-1.8) | 1.4 (1.0-2.0) | 1.9 (1.4-2.5) | 1.4 (1.0-1.9) | 1.5 (1.1-1.9) | 1.7 (1.3-2.3) | 2.4 (1.9-3.0) | 3.3 (2.8-4.0) | 3.2 (2.6-3.8) | 3.5 (2.9-4.1) |

^a^ Rates are per 100 000 and age-adjusted to the 2000 US Standard Population standard.

CI, confidence interval; mPCa, metastatic prostate cancer

# **S4 Table. Non-Hispanic Blacks to non-Hispanic Whites IRRs for bone metastasis and visceral metastasis**

| **Metastasis**  **involvement** | **Years** | **Aged 45-74 years** | |  | **Aged ≥75 years** | |
| --- | --- | --- | --- | --- | --- | --- |
|  |  | **IRR** | **95%CI** |  | **IRR** | **95%CI** |
| **Bone** | 2010 | 2.72 | (2.28-3.24) |  | 2.20 | (1.65-2.93) |
|  | 2011 | 2.69 | (2.29-3.17) |  | 2.00 | (1.50-2.65) |
|  | 2012 | 2.74 | (2.33-3.22) |  | 1.81 | (1.38-2.36) |
|  | 2013 | 3.00 | (2.58-3.49) |  | 1.76 | (1.36-2.27) |
|  | 2014 | 2.47 | (2.13-2.86) |  | 1.49 | (1.14-1.94) |
|  | 2015 | 2.33 | (2.01-2.70) |  | 1.52 | (1.19-1.94) |
|  | 2016 | 2.42 | (2.10-2.78) |  | 1.70 | (1.36-2.13) |
|  | 2017 | 2.39 | (2.10-2.73) |  | 1.63 | (1.31-2.03) |
|  | 2018 | 2.49 | (2.19-2.83) |  | 1.48 | (1.19-1.85) |
|  | 2019 | 2.20 | (1.93-2.51) |  | 1.48 | (1.19-1.84) |
| **Visceral** | 2010 | 4.13 | (2.73-6.24) |  | 4.24 | (2.30-7.81) |
|  | 2011 | 3.57 | (2.28-5.59) |  | 1.89 | (0.90-3.99) |
|  | 2012 | 5.14 | (3.45-7.66) |  | 1.74 | (0.83-3.66) |
|  | 2013 | 3.30 | (2.27-4.80) |  | 1.61 | (0.73-3.52) |
|  | 2014 | 3.56 | (2.44-5.19) |  | 3.71 | (2.07-6.65) |
|  | 2015 | 3.40 | (2.39-4.84) |  | 1.71 | (0.93-3.15) |
|  | 2016 | 3.60 | (2.73-4.75) |  | 1.66 | (1.04-2.66) |
|  | 2017 | 2.94 | (2.21-3.90) |  | 2.60 | (1.76-3.85) |
|  | 2018 | 3.18 | (2.42-4.16) |  | 1.69 | (1.04-2.75) |
|  | 2019 | 3.22 | (2.50-4.15) |  | 2.50 | (1.71-3.66) |

CI, confidence interval; IRR, incidence rate ratio; mPCa, metastasis prostate cancer

# **S5 Table. Other races to non-Hispanic Whites IRRs for bone metastasis and visceral metastasis**

| **Metastasis**  **involvement** | **Years** | **Aged 45-74 years** | |  | **Aged ≥75 years** | |
| --- | --- | --- | --- | --- | --- | --- |
|  |  | **IRR** | **95%CI** |  | **IRR** | **95%CI** |
| **Bone** | 2010 | 1.09 | (0.91-1.30) |  | 1.09 | (0.86-1.39) |
|  | 2011 | 0.92 | (0.78-1.10) |  | 1.04 | (0.83-1.32) |
|  | 2012 | 1.05 | (0.89-1.23) |  | 1.05 | (0.86-1.30) |
|  | 2013 | 1.23 | (1.05-1.43) |  | 0.92 | (0.75-1.14) |
|  | 2014 | 1.00 | (0.87-1.15) |  | 0.85 | (0.69-1.04) |
|  | 2015 | 1.03 | (0.89-1.18) |  | 0.86 | (0.71-1.04) |
|  | 2016 | 1.03 | (0.90-1.17) |  | 0.84 | (0.70-1.01) |
|  | 2017 | 0.92 | (0.81-1.05) |  | 0.85 | (0.72-1.02) |
|  | 2018 | 0.94 | (0.83-1.07) |  | 0.94 | (0.80-1.10) |
|  | 2019 | 0.95 | (0.84-1.08) |  | 0.88 | (0.75-1.04) |
| **Visceral** | 2010 | 0.88 | (0.53-1.44) |  | 1.34 | (0.72-2.52) |
|  | 2011 | 1.43 | (0.91-2.23) |  | 1.00 | (0.54-1.84) |
|  | 2012 | 2.29 | (1.56-3.35) |  | 0.85 | (0.45-1.58) |
|  | 2013 | 0.90 | (0.60-1.35) |  | 0.97 | (0.54-1.75) |
|  | 2014 | 1.33 | (0.92-1.94) |  | 0.97 | (0.51-1.83) |
|  | 2015 | 0.90 | (0.60-1.35) |  | 1.12 | (0.71-1.75) |
|  | 2016 | 1.00 | (0.73-1.37) |  | 0.81 | (0.55-1.19) |
|  | 2017 | 1.25 | (0.95-1.65) |  | 1.18 | (0.84-1.64) |
|  | 2018 | 1.12 | (0.85-1.46) |  | 1.20 | (0.85-1.69) |
|  | 2019 | 1.33 | (1.04-1.70) |  | 1.02 | (0.73-1.44) |

CI, confidence interval; IRR, incidence rate ratio; mPCa, metastasis prostate cancer.

**S6 Table. Comparisons of OS and CSS in mPCa with bone and visceral metastasis involvement** **across time periods (2013-2016 v.s. 2010-2012)**

| **Metastasis** | **Age,**  **years** | **Race** | **OS** | |  | **CSS** | |
| --- | --- | --- | --- | --- | --- | --- | --- |
|  |  |  | **HR (95%CI)^a^** | ***p*^a^** |  | **HR (95%CI)^a^** | ***p*^a^** |
| **Bone** | **45-74** | **Non-Hispanic White** | 0.97 (0.95-0.99) | <0.001 |  | 0.95 (0.93-0.97) | <0.001 |
|  |  | **Non-Hispanic Black** | 0.96 (0.93-0.99) | 0.009 |  | 0.94 (0.91-0.97) | <0.001 |
|  |  | **Other** | 0.98 (0.94-1.01) | 0.166 |  | 0.95 (0.92-0.99) | 0.015 |
|  |  |  |  |  |  |  |  |
|  | **≥75** | **Non-Hispanic White** | 0.98 (0.96-1.01) | 0.115 |  | 0.95 (0.93-0.98) | <0.001 |
|  |  | **Non-Hispanic Black** | 1.02 (0.97-1.07) | 0.501 |  | 0.97 (0.91-1.02) | 0.230 |
|  |  | **Other** | 0.98 (0.94-1.03) | 0.473 |  | 0.96 (0.92-1.01) | 0.134 |
|  |  |  |  |  |  |  |  |
| **Visceral** | **45-74** | **Non-Hispanic White** | 0.96 (0.92-1.01) | 0.069 |  | 0.94 (0.90-0.99) | 0.016 |
|  |  | **Non-Hispanic Black** | 0.93 (0.87-0.99) | 0.032 |  | 0.89 (0.83-0.96) | 0.002 |
|  |  | **Other** | 0.92 (0.85-1.00) | 0.053 |  | 0.91 (0.83-1.00) | 0.051 |
|  |  |  |  |  |  |  |  |
|  | **≥75** | **Non-Hispanic White** | 0.93 (0.88-0.97) | 0.003 |  | 0.91 (0.86-0.96) | 0.001 |
|  |  | **Non-Hispanic Black** | 0.92 (0.82-1.03) | 0.132 |  | 0.86 (0.76-0.98) | 0.022 |
|  |  | **Other** | 1.03 (0.93-1.13) | 0.606 |  | 1.01 (0.90-1.12) | 0.889 |

**^a^** Cox proportional hazards regressions adjusted for age and diagnosed year.

CI, confidence interval; CSS, cancer-specific survival; mPCa, metastasis prostate cancer; OS, overall survival.

# Supplementary Figure


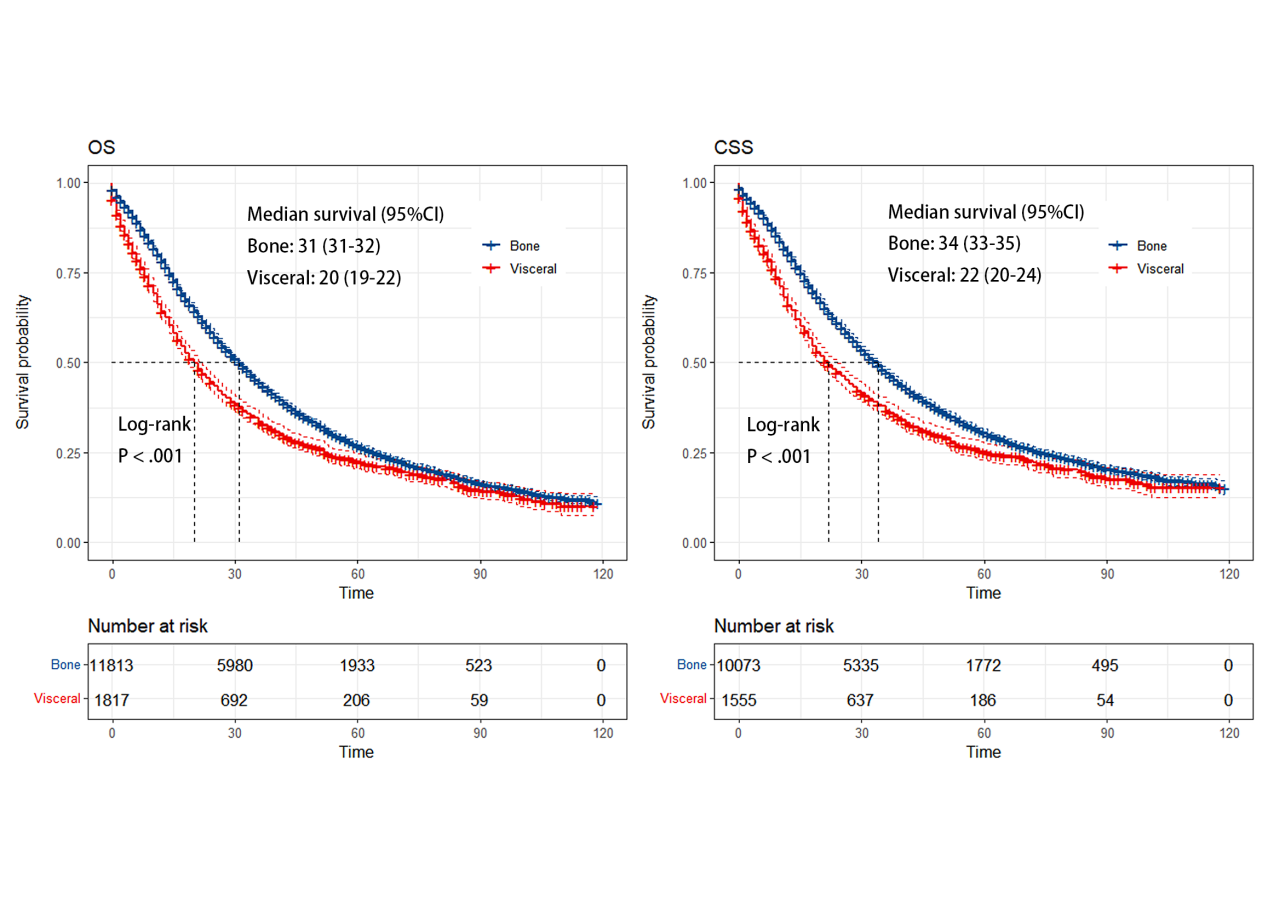


# **S1 Fig.** Kaplan-Meier curves of OS and CSS for mPCa with bone and visceral metastasis involvement.

CI, confidence interval; CSS, cancer-specific survival; mPCa, metastasis prostate cancer; OS, overall survival
